# Cannabidiol in Pharmacoresistant Epilepsy: Clinical Pharmacokinetic Data From an Expanded Access Program

**DOI:** 10.3389/fphar.2021.637801

**Published:** 2021-03-03

**Authors:** Manuela Contin, Susan Mohamed, Margherita Santucci, Monica Anna Maria Lodi, Emilio Russo, Oriano Mecarelli, CBD LICE Italy Study Group

**Affiliations:** ^1^ IRCCS Istituto delle Scienze Neurologiche di Bologna, Bologna, Italy; ^2^ Department of Biomedical and Neuromotor Sciences, University of Bologna, Bologna, Italy; ^3^ IRCCS Istituto Delle Scienze Neurologiche di Bologna, Child Neuropsichiatry, Bologna, Italy; ^4^ Department of Neuroscience, Pediatric Neurology Unit and Epilepsy Center, “Fatebenefratelli e Oftalmico” Hospital, Milano, Italy; ^5^ Science of Health Department, School of Medicine, University Magna Graecia, Catanzaro, Italy; ^6^ Department of Human Neurosciences, Sapienza University, Rome, Italy

**Keywords:** cannabidiol, pharmacokinetics, antiseizure medication, epilepsy, Dravet syndrome, Lennox–Gastaut syndrome

## Abstract

**Background and Aim:** Data on the clinical pharmacokinetics of cannabidiol (CBD) are scanty. We explored the effect of demographic and clinical variables on plasma concentrations of purified CBD in patients with Dravet (DS) and Lennox–Gastaut syndrome (LGS).

**Methods:** The study design was an open, prospective, multicenter expanded access program (EAP). Venous blood samples were drawn from patients between 8 and 9 am, before the CBD morning dose, 12 h apart from the last evening dose, and then 2.5 h after their usual morning dose.

**Results:** We collected 127 plasma samples (67-morning pre-dosing and 60 post-dosing) from 43 patients (24 females, 19 males), 27 with LGS and 16 with DS. Mean ± standard deviation age was 26 ± 15 years. Duration of CBD treatment averaged 4.2 ± 2.9 months at 13.2 ± 4.6 mg/kg/day. CBD median trough plasma concentration was 91 ng/ml; it doubled to 190 ng/ml 2.5 h post-dosing (*p* < 0.001). Cannabidiol trough plasma concentrations were linearly related to daily doses (r = 0.564, *p* < 0.001). Median trough CBD plasma concentration-to-weight-adjusted dose ratio (C/D) was 32% higher (*p* < 0.02) in plasma samples from subjects aged 18 and over than in those under 18. Sex and concomitant antiseizure medications (ASMs) were not associated with significant variations in CBD C/D, but caution is required due to the potential influence of confounders.

**Conclusion:** These are the first data on CBD pharmacokinetics in children and adults with LGS or DS in a real-world setting. The most relevant finding was the higher CBD C/D in adults. In practice, reduced weight-normalized doses might be required with aging to achieve the same CBD plasma levels.

## Introduction

Cannabidiol (CBD) is a nonpsychoactive cannabis-derived compound claimed to possess a variety of pharmacological properties (Amin and Ali, 2019). It is currently being investigated in the treatment of several disorders (Fraguas-Sánchez and Torres-Suárez, 2018), including epilepsy (Franco and Perucca, 2019). Despite the huge number of published studies, both clinically controlled and observational data on the pharmacokinetics of CBD are scanty (Millar et al., 2018). A recent review on CBD dosing in clinical populations, examining 35 studies in 13 different medical contexts, pinpointed that none provided CBD plasma concentrations (Millar et al., 2019).

CBD shows challenging pharmacokinetic characteristics, including very low and variable oral bioavailability and high drug-drug interaction potential (Franco and Perucca, 2019; Landmark and Brandl, 2020; Lattanzi et al., 2020a; Patsalos et al., 2020; Perucca and Bialer, 2020). Published data, mostly from healthy volunteers, show remarkable intersubject variability in CBD plasma concentrations after oral dosing (Millar et al., 2019). No data are available on the potential effects of variables such as age and sex on CBD bioavailability, and knowledge of the effects of concomitant therapies on CBD plasma levels is limited (Franco and Perucca, 2019; Landmark and Brandl, 2020).

A highly purified plant-based form of oral CBD formulation was approved by the United States (US) Food and Drug Administration (FDA) in 2018 and the European Medicines Agency (EMA) in 2019 for the treatment of seizures associated with Dravet (DS) and Lennox–Gastaut syndrome (LGS).

We aimed to explore the effect of dose, age, sex, and concomitant antiseizure medications (ASMs) on steady-state plasma concentrations of CBD in a cohort of patients with highly treatment-resistant DS and LGS receiving this FDA/EMA-approved oral formulation of CBD in the context of an expanded access program (EAP) in Italy. Data were also collected on the potential correlation between CBD plasma concentrations and evidence of both tolerability and seizure control.

## Materials and Methods

### EAP Study Design and Patients

The study design was an open, prospective, multicenter EAP. Thirty Italian epilepsy centers were involved in the study. Inclusion and exclusion criteria for patients’ enrollment are reported in Supplementary Table S1. The study protocol was approved by each site (described in MD September 07, 2017, published in the Official Gazette on November 2, 2017), and written informed consent was obtained from patients or parents/caregivers. Overall data collection was approved by the Ethics Committee “Regione Calabria Area Centro”, Catanzaro (Italy), protocol number 115/19.

During a 4-week baseline period, diaries of all countable seizures were provided by patients and/or parents/caregivers. Afterward, patients received an oral solution of purified CBD (100 mg/ml; Epidyolex GW Research Ltd.), at a starting dosage ranging from 2 to 5 mg/kg/day up to a maximum of 18–25 mg/kg/day.

Follow-up visits to assess seizure control were programmed at 3, 6, 9, and 12 months. Patients with a percentage change in seizure frequency ≥50 compared to a 4-week baseline were classified as responders. Percentage change in seizure frequency for each patient was calculated as [(seizure frequency per 28 days) − (seizure frequency at baseline)]/(seizure frequency at baseline) × 100. Assessment of adverse effects (AEs) and clinical laboratory parameters, including liver tests, was performed at baseline, after 2 weeks, 1, 3, and 6 months of treatment, and then periodically.

Concomitant ASMs were recorded at baseline and during the treatment period. CBD and ASMs doses modification, as well as adding/removing coadministered ASMs, were allowed as clinically indicated.

The collection of clinical data was harmonized among different centers by adopting a standardized case report form.

### Procedures for CBD Plasma Specimen Collection and Quantitation

Inclusion criteria for CBD plasma specimen collection and quantitation were chronic CBD therapy for at least 1 month and no change in dosage of CBD or concomitant ASMs over the preceding 4 weeks. Venous blood samples (3 ml) were drawn from patients between 8 and 9 am, 12 h apart from the last evening dose, and 2.5 h after ingestion of their usual morning dose, taken after breakfast (basically, milk and biscuits for children; milk, or milk and coffee, or coffee, or tea with a pastry for adults). Some patients were sampled on different occasions during their follow-up.

Blood samples were transferred into heparinized tubes and immediately centrifuged at 1,500 × g for 10 min, at 4°C. Plasma was separated and stored at −80°C until analysis, within 6 months from the collection (Andrenyak et al., 2017). Plasma concentrations of CBD were measured by ultra-high-pressure liquid chromatography-mass spectrometry (Dulaurent et al., 2014). All the analyses were performed at the Laboratory of Clinical Neuropharmacology of the Institute of Neurological Sciences of Bologna. The lower limit of quantification (LLOQ) and limit of detection (LOD) were 0.5 and 0.2 ng/ml, respectively. Intra- and interassay imprecision and inaccuracy were ≤15%.

### Data and Statistical Analysis

The sample size was based on patient’s enrollment on each study site and not precalculated. The main study outcome was morning trough CBD plasma concentration-to-weight-adjusted daily dose ratio (C/D) [(ng/ml)/(mg/kg/day)].

ASM comedications were classified as strong enzyme inducers (I), including carbamazepine (CBZ), phenobarbital, and phenytoin (PHT); not strong enzyme inducers/not inhibitors (notI/notInhib), such as brivaracetam, clobazam (CLB), felbamate, lacosamide, lamotrigine, levetiracetam, oxcarbazepine, perampanel, topiramate, rufinamide, zonisamide; and enzyme inhibitors (Inhib), stiripentol (STP), and valproic acid (VPA).

The statistical significance of differences between the two groups was assessed by the Student’s *t*-test or the Mann-Whitney rank-sum test, whenever appropriate. Intrasubject comparisons were performed by the paired *t*-test or the signed-rank test. Correlations between variables were assessed by Pearson’s product-moment coefficient. Clinical variables distribution was compared between patients’ subgroups by chi-square test. Comparisons of CBD C/D ratios among ASM comedication subgroups were carried out by one-way analysis of variance (ANOVA). Pairwise comparisons were performed by the Holm-Sidak method when ANOVAs indicated a significant difference among subgroups. Significance was set at *p* < 0.05. Analyses were carried out by SigmaPlot 13.0 software (Systat Software, San Jose, CA, United States).

## Results

From December 2018 to December 2019, a total of 110 patients were enrolled in the EAP, 93 with complete data available. Between January 29, 2019, and March 19, 2020, we collected 127 plasma samples (67-morning pre-dosing and 60 post-dosing) from 43 patients (24 females, 19 males), 27 with LGS and 16 with DS, enrolled by 13 clinical centers. Mean ± standard deviation (SD) age was 26 ± 15 years (range 5–56 years, <18 years, n = 17). Duration of CBD treatment averaged 4.2 ± 2.9 months (range 1–12 months) at a mean daily dose of 13.2 ± 4.6 mg/kg (range 4.6–22.8 mg/kg/day), in two divided doses (approximately 8 am–8 pm) in all subjects.

**FIGURE 1:**
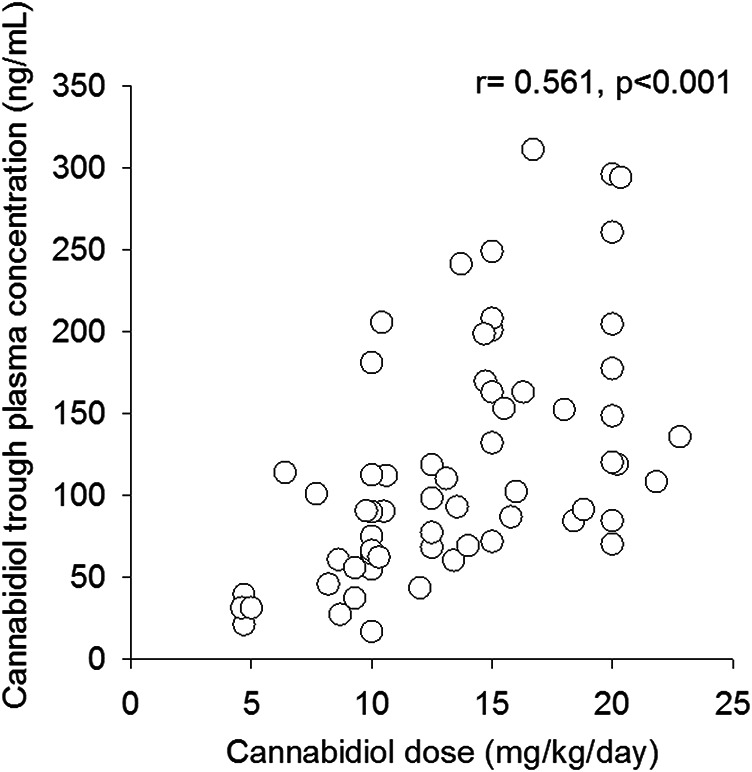
Correlation between cannabidiol trough plasma concentrations and related weight-adjusted daily doses (n = 67).

**FIGURE 2:**
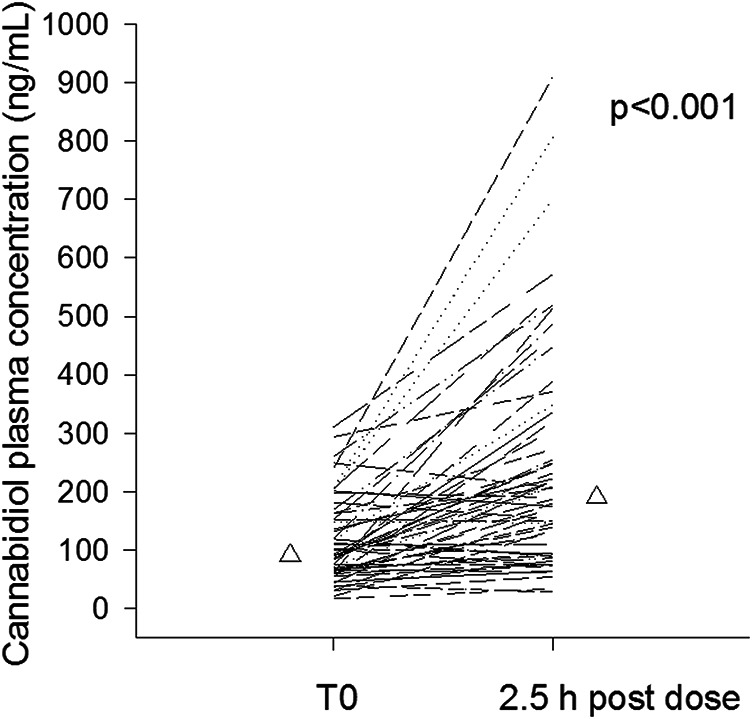
Intrasubject cannabidiol morning trough and 2.5 post-dosing plasma concentrations of cannabidiol (n = 60). Median values are represented by triangles.

CBD median trough plasma concentration was 91 ng/ml (25–75%, 65–153 ng/ml); overall CBD trough plasma concentrations were linearly related to daily doses (r = 0.564, *p* < 0.001, Figure 1). Median trough drug plasma levels doubled to 190 ng/ml (95–322 ng/ml) 2.5 h post-dosing, *p* < 0.001 (Figure 2). Intrasubject CBD concentration-dose relationship obtained in a subset of patients sampled on different occasions during the follow-up is depicted in Supplementary Figure S1. Values of plasma CBD increased almost proportionally with dose in the majority of subjects.

**FIGURE 3:**
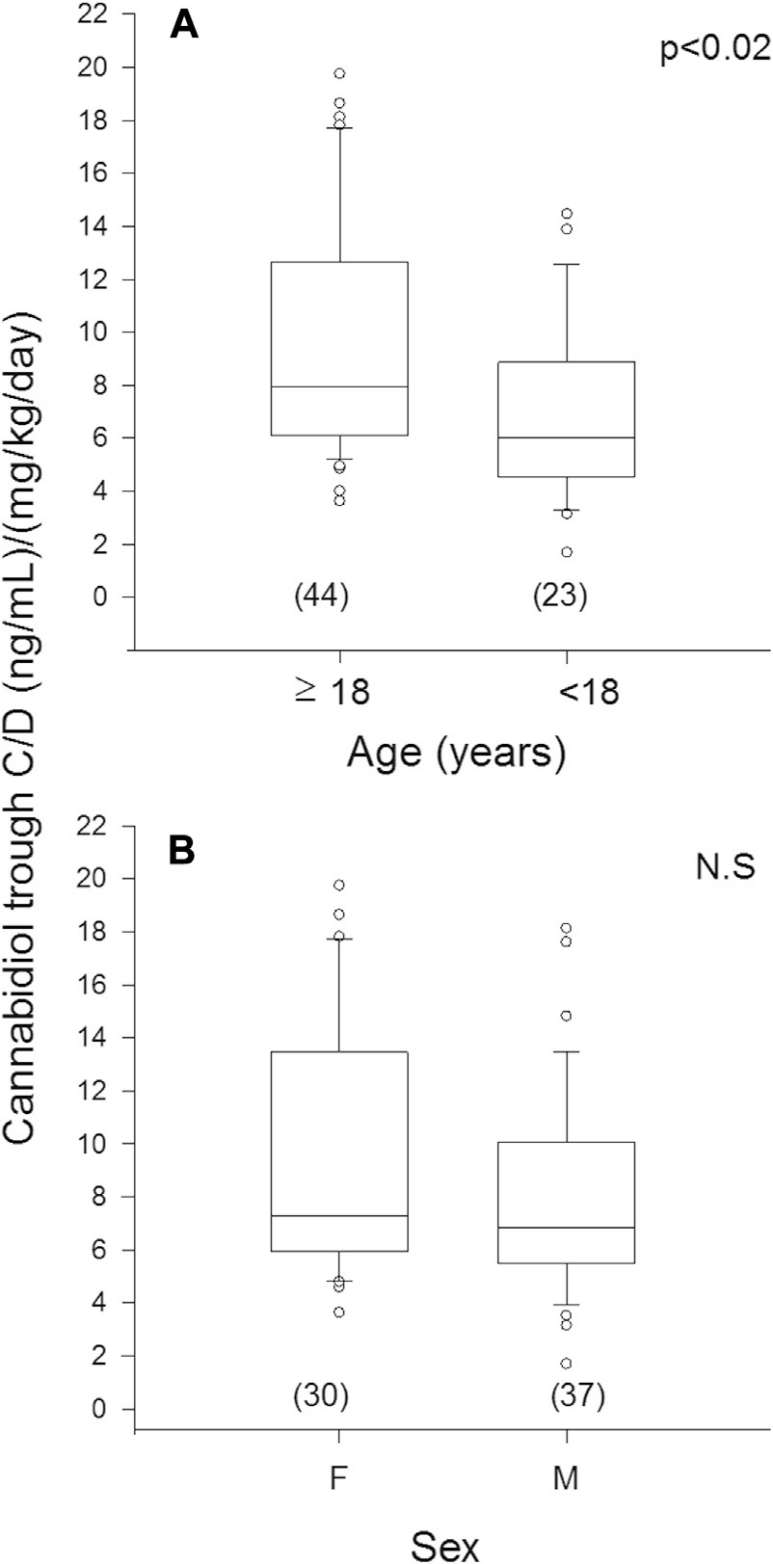
Plasma concentration to weight-adjusted daily dose ratio of cannabidiol (C/D) from patients’ specimens (n) grouped by age **(A)** and sex **(B)**. Box plots depict the range between the 25^th^ and 75^th^ percentiles of the data. The horizontal line marks the median value; capped bars indicate 10^th^–90^th^ percentiles. Black circles represent outlying values. *p*, significance of comparison between age and sex groups by Mann-Whitney rank sum test; N.S., not significant (*p* ≥ 0.05).

Median trough CBD C/D ratio was 32% higher in plasma samples from patients ≥18 years (mean age 35 ± 11 years, range 18–56 years) compared with those <18 years (10 ± 4 years, range 5–17 years): 7.97 vs. 6.02, *p* < 0.02 (Figure 3A). The two age groups were comparable for sex and ASM cotherapies (Supplementary Table S2A).

No significant difference in median trough CBD C/D ratio was observed in plasma samples from males (6.84) vs. females (7.29) (Figure 3B). The female subgroup was significantly older than the male one (*p* < 0.001); strong inhibitor ASMs were more frequently coprescribed in males (Supplementary Table S2B).

**FIGURE 4:**
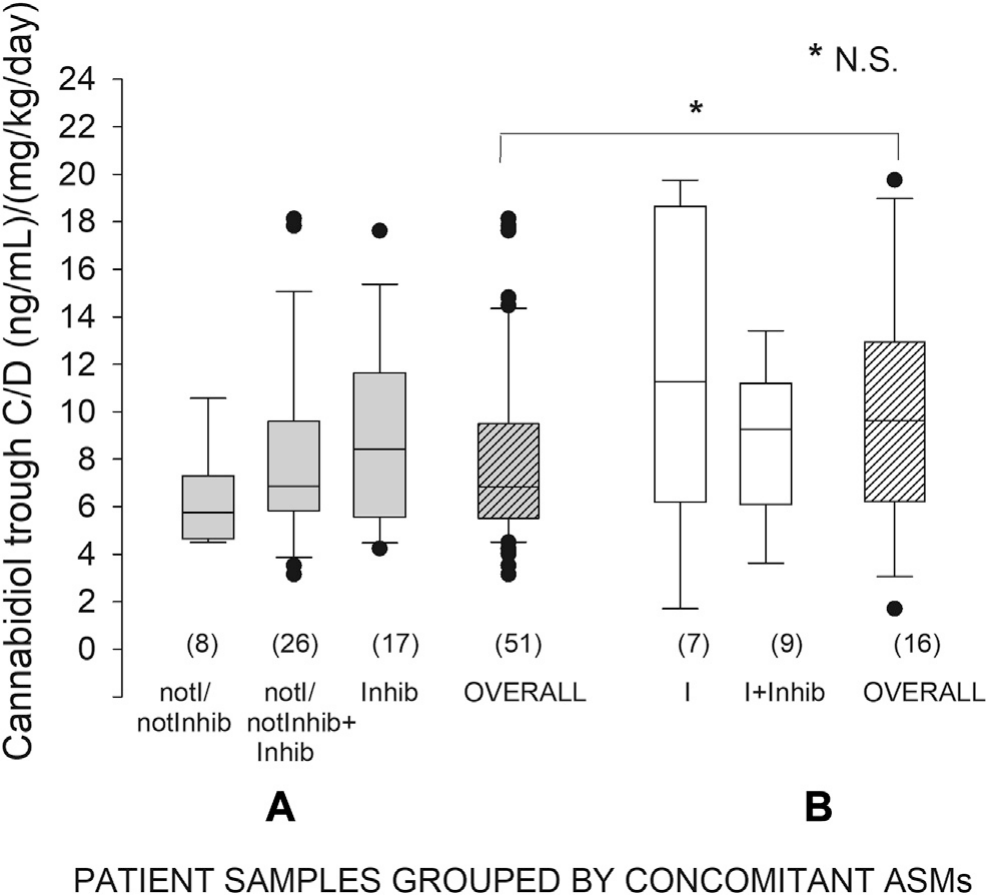
Plasma concentration to weight-adjusted daily dose ratio of cannabidiol (C/D) from patients’ specimens (n) grouped by concomitant antiseizure medication (ASM): **(A)** patients not receiving strong inducers; **(B)** patients receiving strong inducers. Box plots depict the range between the 25^th^ and 75^th^ percentiles of the data. The horizontal line marks the median value; capped bars indicate 10^th^–90^th^ percentiles. Black circles represent outlying values. *p*, significance of comparison between the two overall treatment groups by Mann-Whitney rank sum test; N.S., not significant (*p* ≥ 0.05). NotI/notInhib, not strong enzyme inducers/not inhibitors; I, strong enzyme inducers; Inhib, enzyme inhibitors.

As far as concomitant ASMs are concerned, 7 samples were associated with I prescription, 9 with I + Inihib, 8 with notI/notInhib, 26 with notI/notInhib + Inhib, and 17 with Inhib. The name, number, and daily doses of concomitant ASMs are specified in Supplementary Table S3. From separate analyses, median CBD C/Ds were comparable among notI/notInhib, notI/notInhib + Inhib, and Inhib ASM subgroups, which were pooled together for subsequent analyses. Similarly, I and I + Inhib ASM subgroups did not differ and were pooled together. No significant difference was observed in median C/D ratios of CBD segregated in two main categories, with (n = 16) and without (n = 51) concomitant strong enzyme-inducing ASMs: 9.63 vs. 6.84 (Figure 4). These two groups were comparable for sex distribution but different for age, which was significantly older in patients cotreated with strong enzyme inducers (38 ± 14 vs. 22 ± 13 years, *p* < 0.001).

Thirty-six plasma samples (54%) were matched to CBD responder patients. Both median CBD plasma concentrations (106 vs. 87 ng/ml) (Supplementary Figure S2A) and C/Ds (7.69 vs. 6.33) (Supplementary Figure S2B) did not differ between responders and nonresponders. Clinical and therapeutic characteristics were comparable between these two subgroups, except for age and CBD treatment duration, which were, respectively, older (*p* < 0.01) and shorter (*p* < 0.02) in responders (Supplementary Table S4A). In particular, as far as ASM cotherapy is concerned, distribution of CLB cotherapy did not differ between the two groups (16 out of 36 from responder plasma samples and 20 out of 31 from nonresponders, *p* = 0.162).

Twenty-nine plasma samples (43%) were associated with AE reports. They included mainly gastrointestinal disorders (32%), namely, appetite loss, diarrhea, followed by somnolence (18%), increase in transaminase levels (13%), and behavioral changes (11%), such as agitation or irritability. Increase in transaminase levels was observed in all patients receiving VPA.

No difference was found either in CBD plasma concentrations (93.1 vs. 90.4) (Supplementary Figure S2A) or in C/D ratio (7.87 vs. 6.74) (Supplementary Figure S2B) between the two groups with or without evidence of CBD-related AEs. The AE subgroup was characterized by older age (*p* < 0.02), lower CBD daily dose (*p* < 0.01), shorter treatment duration (<0.008), and higher frequency of strong enzyme-inducing ASM cotherapy (*p* < 0.008) (Supplementary Table S4B). Frequency of CLB cotreatment was similar between the two subgroups (14 out of 29 with EAs vs. 22 out of 38 without AEs, *p* = 0.593).

## Discussion

To the best of our knowledge, these results are the first on the effect of demographic and clinical variables on CBD plasma concentrations in *“real”* children and adults with LGS and DS. In our cohort, plasma concentrations of CBD were linearly related to matched daily dose, expressed as mg/kg/day, over a range of 5–23 mg/kg/day. This result is in keeping with findings obtained in pediatric patients by Devinsky et al. (2018a), over a 5–20 mg/kg/day dose range, and Wheless et al. (2019), over 5–40 mg/kg/day doses, and in healthy adult volunteers after multiple doses (750–1,500 mg/day) (Taylor et al., 2018). Plasma concentration-dose linearity is an important drug characteristic in clinical practice, as it may help physicians in patients’ dose adjustments. However, it was characterized by a large, up to 10-fold, intersubject variation in drug trough plasma concentrations at a given dosage. High intersubject variability in CBD bioavailability has been reported in patients (Devinsky et al., 2018a; Wheless et al., 2019) and in healthy subjects (Taylor et al., 2018; Crockett et al., 2020), partly ascribed to CBD incomplete oral absorption and large pre-systemic elimination (Perucca and Bialer, 2020). Moreover, food intake, especially high fat/high caloric meals, has a marked effect on CBD exposure, increasing drug bioavailability up to 4-5-fold (Taylor et al., 2018; Crockett et al., 2020). All our patients took their morning CBD dose in a fed state, but breakfast type was not standardized.

Notably, up to 8-fold fluctuations in intrasubject CBD plasma concentrations were observed in most patients’ samples between the morning trough and 2.5 h post-dosing, in line with previous evidence (Devinsky et al., 2018a). Reported times to peak of plasma CBD oral formulations are highly variable, mostly in the range of 1–4 h (Millar et al., 2018). We established the time of post-dosing blood sampling based on the clinical trial of Devinsky et al. (2018a), using the same CBD oral solution.

A novel finding was the significant effect of age on median trough CBD C/D ratio, which was higher in subjects aged 18 and over than in those under 18. Cannabidiol undergoes both an extensive first-pass effect and metabolism in the liver (Franco and Perucca, 2019; Perucca and Bialer, 2020), and age-mediated reduction in both these processes may partly explain this observation (Van den Elsen et al., 2014). The only data reported so far on the potential influence of age on CBD pharmacokinetics were confined within a cohort of pediatric patients (Wheless et al., 2019). At any given dosage, plasma CBD concentrations were lower in infants (aged 1 to <2 years) compared to children (2 to <12 years) and adolescents (12 ≤17 years).

Sex did not affect median trough C/D ratio of CBD in our patients. This observation should be considered cautiously as potentially influenced by the older age of the women group. Of note, no study has explored so far the potential differences between males and females in cannabinoids pharmacokinetics (Millar et al., 2018), which might contribute to observed sex-dependent differences in some of their effects (Fattore and Fratta, 2010). From a theoretical point of view, it has been hypothesized that the larger percentage of body fat in women might result in an increased volume of distribution of lipophilic compounds such as CBD, with a higher proportion of drug concentration sequestered in fat tissue and reduced drug plasma concentrations (Fattore and Fratta, 2010). This should also be linked to the peculiar half-life of CBD being initially shorter and then longer according to the possibility of compartmentalization of the drug in some not defined deep compartments (e.g., adipose tissue) (Lattanzi et al., 2020a).

No significant differences emerged in CBD C/D ratio from patients’ samples grouped based on metabolism inducing or inhibiting properties of concomitant ASMs. The interpretation of these results is limited by the small sample size per ASM cotherapy groups coupled with high within-group intersubject variability in CBD C/Ds, especially in patients taking strong enzyme inducers. Moreover, the influence of confounders, such as older age of the subgroup on inducers, cannot be ruled out. CBD is metabolized by the cytochrome P450 isoenzyme CYP2C19 to the active metabolite 7-hydroxy-CBD and further to inactive metabolites through CYP3A4 and uridine 5′-diphospho-glucuronosyltransferases (UGTs) (Landmark and Brandl, 2020). Enzyme-inducing ASMs, especially CBZ and PHT, would be expected to reduce CBD C/D ratio (Franco and Perucca, 2019), but no formal study has explored so far this potential interaction. Data on the effect of concomitant ASMs on CBD pharmacokinetics are scanty. From a phase I, open-label healthy volunteer trial (Morrison et al., 2019), concomitant intake of metabolism inhibitors such as STP (750 mg b.i.d., for 14 days) and VPA (500 mg b.i.d., for 14 days) had no significant effect on CBD bioavailability; 7-hydroxy-CBD exposure was decreased by 29% by STP, but the underlying mechanism is unknown. Clobazam (5 mg b.i.d., for 21 days) did not affect CBD exposure, while 7-hydroxy-CBD increased 1.5-fold, possibly by inhibition of UGTs (Morrison et al., 2019).

Dose-dependency for both efficacy and tolerability was not evidenced by our data. Attempts to find out a relationship between CBD plasma concentrations and both seizure control and AEs yielded no significant results. Plasma CBD values associated with therapeutic efficacy or AEs were overlapping. These findings might partly reflect high intersubject variability in CBD bioavailability, patients’ different clinical characteristics, and the heterogeneous contribution of different types and doses of concomitant ASMs.

The observed 54% responder rate was in line with the 38–52% previously reported in open-label studies (Devinsky et al., 2016; Thiele et al., 2019), a EAPs (Szaflarski et al., 2018; Laux et al., 2019) and randomized controlled trials (Devinsky et al., 2018b) involving patients with pharmacoresistant epilepsies treated with the same oral solution of purified CBD. Of note, distribution of CLB cotherapy did not differ between responders and nonresponders, in line with the evidence coming from randomized controlled studies that CBD has antiseizure activities irrespective of CLB coadministration (Bialer and Perucca, 2020; Lattanzi et al., 2020b).

The AE rate of 43% was lower than 79–94% reported in the abovementioned studies; the most common registered AEs, appetite loss, diarrhea, and somnolence were in line with the literature.

All these observations should be taken with caution due to the limited number of patients, the uncontrolled design of EAP protocol, and possible intersite variability in reporting methods, among others. Further studies in larger cohorts of patients are needed to confirm these findings.

## Conclusion

We provide for the first time a picture of CBD pharmacokinetics in patients with LGS and DS under an EAP, a study condition that is closer to “*real*” patients compared with controlled clinical trials. The most relevant finding was the evidence of a significant increase in CBD plasma concentrations with aging. Age may be added to the variables contributing to the wide intersubject variability observed in plasma CBD at the same dosages. From a practical point of view, reduced weight-normalized doses might be required with aging to achieve the same CBD plasma levels.

## CBD LICE Italy Study Group

**Francesca Bisulli**, IRCCS Istituto delle Scienze Neurologiche di Bologna, Epilepsy Center (Reference Center for Rare and Complex Epilepsies–EpiCARE), Bologna, Italy, and Department of Biomedical and Neuromotor Sciences, University of Bologna, Bologna, Italy; **Antonella Boni**, IRCCS Istituto delle Scienze Neurologiche di Bologna, Child Neuropsichiatry, Bologna, Italy; **Gaetano Cantalupo**, Child Neuropsychiatry, Department of Surgical Sciences, Dentistry, Gynecology and Pediatrics, University of Verona, Verona, Italy; **Elisabetta Cesaroni**, Child Neurology and Psychiatry Unit, G. Salesi Children’s Hospital-University of Ancona, Ancona, Italy; **Antonietta Coppola**, Department of Neuroscience, Reproductive and Odontostomatological Sciences, Epilepsy Centre, University of Naples Federico II, Naples, Italy; **Carlo Di Bonaventura**, Neurology Unit, Department of Human Neurosciences, “Sapienza” University, Rome, Italy; **Anna Fetta**, Child Neurology and Psychiatry Unit, Department of Medical and Surgical Sciences (DIMEC), S. Orsola Hospital, University of Bologna, Bologna, Italy; **Angela La Neve**, Department of Basic Medical Sciences, Neurosciences and Sense Organs, University of Bari, Bari, Italy; **Sara Matricardi**, Child Neurology and Psychiatry Unit, G. Salesi Children's Hospital-University of Ancona, Ancona, Italy; **Roberto Michelucci**, IRCCS Istituto delle Scienze Neurologiche di Bologna, Unit of Neurology, Bellaria Hospital, Bologna, Italy; **Amanda Papa**, Child Neuropsychiatry Department, Maggiore della Carità University Hospital, Novara, Italy; **Nicola Pilolli**, Department of Basic Medical Sciences, Neurosciences and Sense Organs, University of Bari, Bari, Italy; **Patrizia Pulitano**, Department of Human Neurosciences, Sapienza University, Rome, Italy; **Francesca Ragona**, Department of Pediatric Neuroscience, Fondazione IRCCS Istituto Neurologico Carlo Besta, Milano, Italy, member of ERN EpiCARE; **Paola Russo**, Department of Neuroscience, Reproductive and Odontostomatological Sciences, Epilepsy Centre, University of Naples Federico II, Naples, Italy; **Pasquale Striano**, Pediatric Neurology and Muscular Diseases Unit, IRCCS “G. Gaslini” Institute, Genoa, Italy, and Department of Neurosciences, Rehabilitation, Ophthalmology, Genetics, Maternal and Child Health, University of Genova, Genova, Italy; **Lilia Volpi**, IRCCS Istituto delle Scienze Neurologiche di Bologna, Unit of Neurology, Bellaria Hospital, Bologna, Italy; **Claudio Zucca**, Clinical Neurophysiology Unit, Scientific Institute, IRCCS Eugenio Medea, Bosisio Parini, Lecco, Italy.

## Data Availability

The raw data supporting the conclusions of this article will be made available by the authors, without undue reservation.
