# Supplementary material for: Cannabidiol in Pharmacoresistant Epilepsy: Clinical Pharmacokinetic Data From an Expanded Access Program
Source: Front Pharmacol. 2021 Mar 3;12:637801. doi: 10.3389/fphar.2021.637801 (PMC7966506; doi:10.3389/fphar.2021.637801)
Supplement: Supplementary file 1 [file datasheet1.docx]

Supplementary Material

**Table 1.** Inclusion and exclusion criteria to access the Italian expanded access program for

cannabidiol

**_______________________________________________________________________________**

**Inclusion criteria**

1. Aged 2 years and above;
2. Dravet syndrome or Lennox-Gastaut syndrome diagnosis;
3. Currently taking between 1 and 4 other ASMs, with a stable antiseizure treatment for the previous 4 weeks (including ketogenic diet and vagus nerve stimulation);
4. Lack of seizure control due to failed treatment with 2 or more prior ASMs
5. Written informed consent provided by the participant and/or parent(s)/caregiver(s).

**Exclusion criteria**

1. Pregnancy, lactation, and risk of pregnancy
2. Clinically significant unstable medical or psychiatric conditions that may place the patient’s safety at risk;
3. Clinically significant liver disease or serum aminotransferase (ALT or AST) >3 times the upper limit of the normal range or total bilirubin > 2 times the upper limit of the normal range or international normalized ratio (INR) > 1.5.
4. Known or suspected intolerance or hypersensitivity to cannabinoids or any of the excipients of the medicinal product such as sesame oil;
5. Stable felbamate dosing < 1 year
6. Current use (or use in the previous 2 months) of recreational or medicinal cannabis, or synthetic cannabinoid-based medications.
7. Alcohol abuse < 2 years before cannabidiol initiation

**________________________________________________________________________________**

**Figure 1**. Intrasubject relationship between cannabidiol trough plasma concentrations and

matched daily doses on different occasions during the follow-up

|  | Age groups | |  |
| --- | --- | --- | --- |
| Variable | ≥ 18 years  (n=44) | < 18 years  (n=23) | p value |
| Age (years) | 35±11 | 10±4 | <0.001 |
| Sex (f/m) | 22/22 | 7/16 | 0.202 |
| Weight (kg) | 69±17 | 34±12 | <0.001 |
| CBD dose (mg/kg/day) | 13.0±4.5 | 13.7±4.9 | 0.577 |
| Syndrome (LGS/DS) | 35/9 | 7/16 | <0.001 |
| Type of ASM cotherapy  Strong enzyme inducers  Not strong inducers/not strong inhibitors  Enzyme inhibitors | n= 13  n= 22  n= 33 | n= 3  n=12  n=19 | 0.139  0.870  0.938 |

**Table 2a.** Clinical characteristics associated with patients’ plasma samples grouped by age

**Table 2b.** Clinical characteristics associated with patients’ plasma samples grouped by sex

|  | Sex | |  |
| --- | --- | --- | --- |
| Variable | Females  (n=30) | Males  (n=37) | p value |
| Age | 33±15 | 21±12 | <0.001 |
| Weight (kg) | 63±23 | 52±22 | 0.05 |
| CBD dose (mg/kg/day) | 14.4±4.1 | 12.4±4.9 | 0.08 |
| Syndrome (LGS/DS) | 22/8 | 20/17 | 0.171 |
| Type of ASM cotherapy  Strong enzyme inducers  Not strong inducers/not strong inhibitors  Enzyme inhibitors | n= 11  n= 17  n= 19 | n= 5  n=17  n=33 | 0.055  0.531  <0.03 |

Data are expressed as mean±SD; f, females; m, males; CBD, cannabidiol; LGS, Lennox Gastaut Syndrome; DS, Dravet Syndrome; ASM, antiseizure medication

**Table 3.** Concomitant antiseizure medication associated with patients’ plasma samples

| Strong enzyme inducers,  dose (mg/day) (n) | Not strong inducers/  not strong inhibitors,  dose (mg/day) (n) | Strong enzyme inhibitors,  dose (mg/day) (n) |
| --- | --- | --- |
| Carbamazepine, 1100±200 (6)  Phenobarbital, 106±32 (8)  Phenytoin, 300 (2) | Brivaracetam, 93±60 (5)  Clobazam, 18±9 (36)  Clonazepam, 2 (6)  Ethosuximide 550±204 (4)  Felbamate 1515±810 (4)  Lacosamide, 361±157 (8)  Lamotrigine, 310±166 (12)  Levetiracetam, 1000 (3)  Nitrazepam, 3.7±3 (5)  Oxcarbazepine, 1200 (1)  Perampanel, 6 (4)  Rufinamide, 1607±727 (7)  Topiramate, 210±110 (12)  Vigabatrin, 1500 (1)  Zonisamide, 400±173 (5) | Stiripentol, 1125±476 (18)  Valproic acid, 952±385 (42) |

Doses are expressed as mean±SD.

**Table 4a.** Clinical characteristics associated with patients’ plasma samples grouped by

cannabidiol efficacy

| Variable | Responders  (n=36) | Nonresponders  (n=31) | p value |
| --- | --- | --- | --- |
| Sex (f/m) | 19/17 | 11/20 | 0.241 |
| Age (years) | 30±13 | 21±14 | <0.01 |
| CBD dose (mg/kg/day) | 13.2±4.9 | 13.3±4.4 | 0.988 |
| CBD treatment duration (months)* | 3 (1.2-5) | 3 (3-6) | <0.02 |
| Syndrome (LGS/DS) | 24/12 | 18/13 | 0.637 |
| Type of ASM cotherapy  Strong enzyme inducers  Not strong inducers/not strong inhibitors  Enzyme inhibitors | n= 10  n= 17  n= 30 | n= 6  n=17  n=22 | 0.649  0.706  0.359 |

**Table 4b.** Clinical characteristics associated with patients’ plasma samples grouped by cannabidiol

tolerability

|  | Adverse effects | |  |
| --- | --- | --- | --- |
| Variable | Yes  (n=29) | No  (n=38) | p value |
| Sex (f/m) | 14/15 | 16/22 | 0.798 |
| Age (years) | 31±16 | 22±13 | <0.02 |
| CBD dose (mg/kg/day) | 11.6±3.9 | 14.4±4.8 | <0.01 |
| CBD treatment duration (months)* | 3 (2-3) | 3.5 (3-6) | <0.008 |
| Syndrome (LGS/DS) | 21/8 | 21/17 | 0.237 |
| Type of ASM cotherapy  Strong enzyme inducers  Not strong inducers/not strong inhibitors  Enzyme inhibitors | n= 12  n= 12  n= 22 | n= 4  n=22  n=30 | <0.008  0.274  0.996 |

Data are expressed as mean±SD or median* (25-75 percentiles); f, females; m, males; CBD, cannabidiol; LGS, Lennox Gastaut Syndrome; DS, Dravet Syndrome; ASM, antiseizure medication


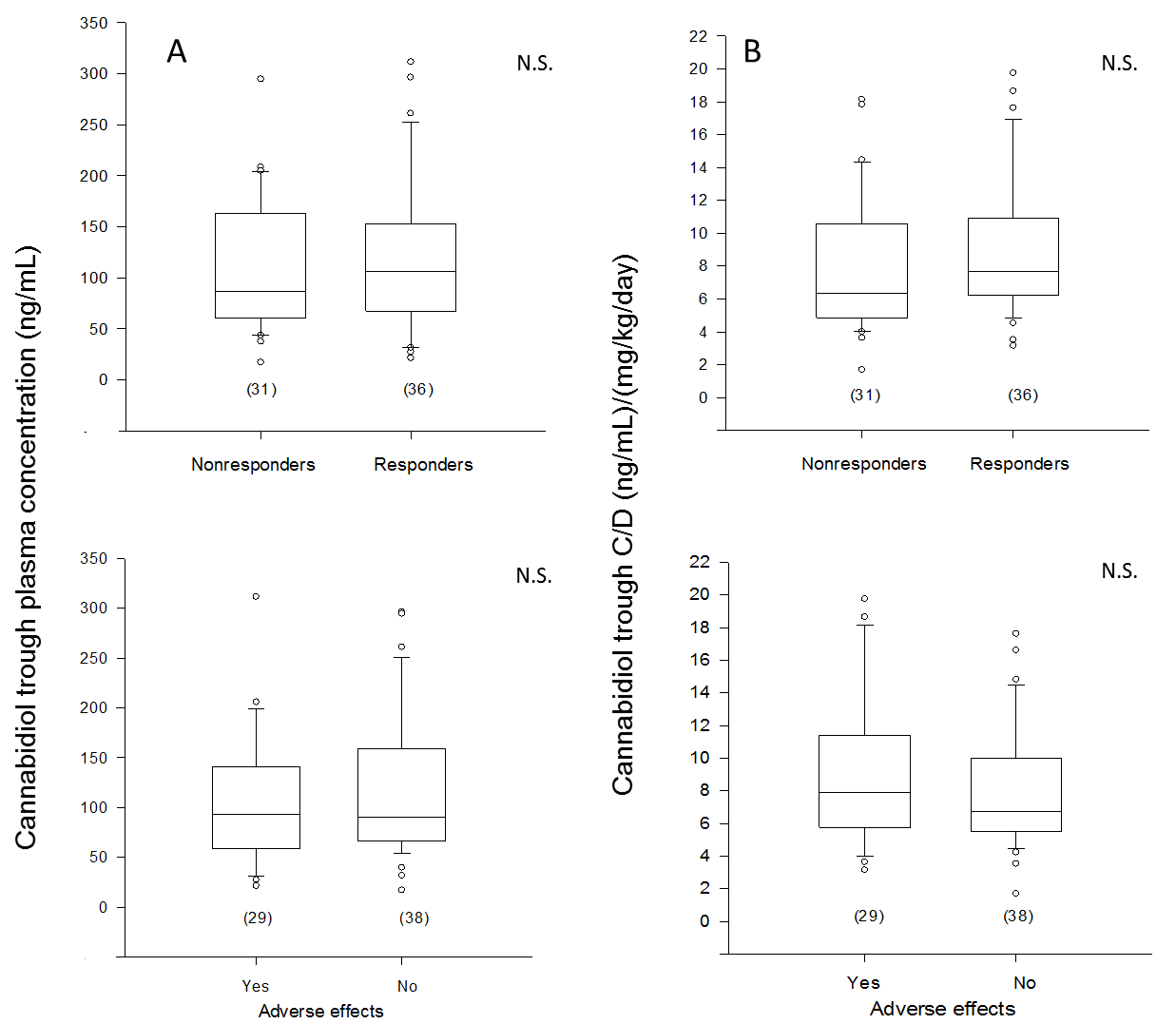


**Figure 2S**. Plasma concentration of cannabidiol (panel A) and matched plasma concentration to

weight-adjusted daily dose ratio (C/D) (panel B) from patients’ specimens (n)

grouped according to cannabidiol efficacy (top) and tolerability (bottom). Box plots

depict the range between the 25th and 75th percentiles of the data. The horizontal line

marks the median value; capped bars indicate 10th-90th percentiles. Black circles

represent outlying values. N.S., not significant (p>0.05), according to Mann-Whitney

rank sum test.
